# Impact of daratumumab on stem cell collection in patients with AL amyloidosis

**DOI:** 10.1186/s12885-026-17227-0

**Published:** 2026-11-03

**Authors:** Joseph Kauer, Tobias Dittrich, Andreas Smeykal, Kiavasch M. N. Farid, Anthony D. Ho, Carsten Müller-Tidow, Anita Schmitt, Sandra Sauer, Ute Hegenbart, Stefan O. Schönland

**Affiliations:** 1 Internal Medicine V, Hematology, Oncology and Rheumatology, Heidelberg University Hospital, Heidelberg, Germany; 2 Molecular Medicine Partnership Unit (MMPU), University of Heidelberg and European Molecular Biology Laboratory (EMBL), Heidelberg, Germany

**Keywords:** AL amyloidosis, Autologous stem cell transplantation, Stem cell mobilization, Daratumumab, Quadruplet induction

## Abstract

**Background:**

Autologous stem cell transplantation following high-dose melphalan is an effective consolidation therapy for the initial treatment of systemic AL amyloidosis. While quadruplet induction therapy involving daratumumab is the standard-of-care, its impact on stem cell yield has not been studied. Furthermore, the relation of stem cell doses and long-term bone marrow function following quadruplet induction therapy remains unclear. It is unknown whether stem cell mobilization with G-CSF and plerixafor is comparable to that achieved with chemotherapy in AL amyloidosis.

**Methods:**

We analyzed the impact of induction and mobilization regimens on stem cell yield in 180 patients using a prospectively maintained database. Descriptive analyses and multivariable logistic regression analyses were performed for collection outcomes. The primary endpoint was overall stem cell collection yield.

**Results:**

The median age at diagnosis was 55 years, with 62% of patients being male. Organ involvement included heart in 48% and kidney in 60% of patients. For induction, 51% of patients received a bortezomib-based regimen (e.g. CyBorD or BorD), 15% received a daratumumab-containing regimen and 27% underwent chemotherapy-free induction therapy. Chemo-free mobilization with G-CSF was performed in 23% of patients, while 39% received cyclophosphamide and dexamethasone (CD) and 33% received cyclophosphamide, adriamycin and dexamethasone (CAD). The total collection after chemo-free mobilization was lower compared to CD and CAD (5.44 × 10⁶ versus 7.29 × 10⁶ versus 9.69 × 10⁶ CD34/kg, p < 0.001). Daratumumab decreased the yield compared to chemotherapy-free induction (5.93 versus 9.24 × 10⁶/kg, p = 0.013) and showed a negative trend compared to bortezomib-based regimen (8.01 × 10⁶/kg, p = 0.147) in univariable analysis. Multivariable logistic regression analysis showed that daratumumab-exposed patients collected stem cell products of > 6.5 × 10⁶/kg less often (Odds ratio (OR) 0.40, p = 0.079). The same was true for patients undergoing mobilization with G-CSF and without plerixafor (OR 0.32, p = 0.012). Stem cell transplantation with > 6.5 × 10⁶/kg might improve short-term and long-term bone marrow function, as reflected by a lower incidence of thrombocytopenia.

**Conclusion:**

Mobilization regimens had a stronger impact on stem cell yield than induction therapies. Stem collection is feasible after DaraCyBorD, however, high stem cell doses should be applied to maintain adequate bone marrow function.

**Supplementary information:**

The online version contains supplementary material available at https://doi.org/10.1186/s12885-026-17227-0.

## Highlights

Quadruplet induction leads to collection of fewer high-dose stem cell products.

Stem cell doses > 6.5 × 10^6^/kg ensure adequate short-term and long-term bone marrow function.

Stem cell mobilization with G-CSF and plerixafor is feasible after DaraCyBorD.

**Supplementary information:**

The online version contains supplementary material available at https://doi.org/10.1186/s12885-026-17227-0.

## Introduction

Amyloid light chain (AL) amyloidosis is a protein deposition disorder with underlying plasma cell dyscrasia. Effective reduction of paraprotein-secreting cell clones, e.g., in the bone marrow, is a prerequisite for organ response in the treatment of AL amyloidosis [1, 2]. As of today, standard induction therapy for fit patients is comprised of the anti-CD38 antibody daratumumab (DARA), proteasome inhibitors such as bortezomib (BTZ), cyclophosphamide (CYCLO) and dexamethasone (DEXA) [3–6]. In eligible patients, high-dose chemotherapy (HDM) followed by autologous stem cell transplantation (ASCT) is an effective consolidation therapy and leads to over 19 years of median overall survival (OS) in patients achieving CR [7–10]. Improved eligibility criteria led to a reduction of treatment-related mortality from 40% to under 5% [9]. Approximately 20% of patients with newly diagnosed AL amyloidosis are eligible for HDM/ASCT according to recent guidelines [4, 8, 11]. While certain centers only treat patients with full-dose melphalan, eligibility may be extended by application of 100 or 140 mg/m^2^ in select patients with renal dysfunction [12]. As a result, patients aged 70–75, those with impaired renal function and status post heart transplant might also be eligible for HDM/ASCT [13–15]. In patients with sufficient response after induction, HDM/ASCT may be deferred without negative impact on prognosis [16, 17]. Response-driven approaches with first-line HDM/ASCT in patients with unsatisfactory response are also feasible [18]. However, the role of autologous transplantation is declining, since quadruplet induction therapy is sufficient to induce long-lasting remission in a proportion of patients [3, 19]. In addition, novel second line therapies such as venetoclax, chimeric antigen receptor T cells (CART), and bispecific antibodies (bsAb) challenge the role of HDM/ASCT [5, 20–22].

Collection of autologous stem cells after peripheral blood mobilization is a prerequisite for HDM/ASCT therapy. Stem cell collection is either performed after mobilization chemotherapy followed by G-CSF application or via chemo-free mobilization using only G-CSF [8, 23, 24]. In the last decade, the CXCR4 antagonist plerixafor improved collection outcomes in multiple myeloma and is increasingly used in AL amyloidosis patients [25–28]. Chemo-free mobilization with G-CSF/plerixafor is standard-of-care in most centers and was adopted by us in 2024. However, we also analyzed stem cell collection after application of either cyclophosphamide and dexamethasone followed by G-CSF (CYCLO/G-CSF) or CYCLO combined with adriamycin and dexamethasone (CAD/G-CSF) as well [8, 23]. Chemomobilization may lead to a higher stem cell yield [23]. While quadruplet induction impairs stem cell collection in multiple myeloma, data on stem cell yields and hematopoietic reconstitution after triplet and quadruplet induction therapy in AL amyloidosis are scarce [29–31]. We here report on stem cell collection and bone marrow function after triplet and quadruplet induction therapies in *n* = 180 AL amyloidosis patients treated at the Amyloidosis Center Heidelberg.

## Methods

### Patients

For this study, *n* = 180 consecutive patients with systemic AL amyloidosis that underwent autologous stem cell mobilization and collection between 2008 and 2024 at the Heidelberg Amyloidosis center were included. Patient characteristics as well as stem cell mobilization and collection parameters were collected from a prospectively maintained database. Patient characteristics are summarized in Table 1. Patients were grouped according to induction therapy: bortezomib-based regimen (e.g., CyBorD, BorD, applied 2007–2022), daratumumab-containing regimen (predominantly Dara-CyBorD, 2018-ongoing), chemotherapy-free induction (only glucocorticoids allowed, 2007–2020). Patients were further grouped regarding the mobilization therapy: Chemo-free mobilization with G-CSF (performed from 2009 to 2023), G-CSF with plerixafor on demand (2024-ongoing), cyclophosphamide and dexamethasone (CYCLO, 2009–2023) or cyclophosphamide, adriamycin, and dexamethasone (CAD, 2008–2015).

**Table 1:** Patient characteristics and PBSC collection metrics. G-CSF, granulocyte colony-stimulating factor; GI, gastrointestinal; IMiD, immunomodulatory drugs; LP, leukapheresis; ; m^2^, square meter; MGUS, monoclonal gammopathy of undetermined significance; MM, multiple myeloma; SD, standard deviation

	*n*	%
**GENERAL INFORMATION**		
**Patient number**,** n**	180	100
**Gender**,** n (%)**		
Male	112	62
Female	68	38
**FIRST DIAGNOSIS**		
**Diagnosis**		
MM	35	19
MGUS	81	45
Smouldering	63	35
Other	1	1
**Median age at diagnosis**,** years (range)**	55	(36–69)
**Mean age at diagnosis**,** years (SD)**	54	7
**Light chain type**,** n (%)**		
Lambda	131	73
Kappa	49	27
**Organ involvement**		
Heart	86	48
Kidney	108	60
Liver	28	16
Peripheral nervous system	21	12
Autonomic nervous system	15	8
Gastrointestinal tract	36	20
Soft tissue	75	42
Median number of involved organs (range)	2	(1–6)
**INDUCTION TREATMENT**		
**First line treatment - exposure to agents**		
Daratumumab-exposed	27	15
Bortezomib-exposed	118	66
Melphalan-exposed	4	2
**Induction group**		
Daratumumab-based (e.g., DaraCyBorD, DaraBorD)	27	15
Bortezomib-based (CyBorD, BorD)	92	51
Chemotherapy-free (No treatment or glucocorticoids)	49	27
Other (e.g., IMiD, melphalan)	12	7
**Median number of induction cycles (range)**	3	(0–15)
**Median time from diagnosis to mobilization (months**,** range)**	6	(0-202)
**PBSC MOBILIZATION/COLLECTION**	n	%
**Mobilisation regimen**		
Cyclophosphamide, adriamycin, dexamethasone (CAD)	59	33
Cyclophosphamide-Dexamethasone	70	39
G-CSF only	41	23
G-CSF + pre-emptive plerixafor	10	6
**Rescue Plerixafor**		
Yes	20	11
No	160	89
**Blood counts prior to mobilization**		
Mean leukocytes /nl (+/- SD)	9	4
Mean Thrombocytes /nl (+/- SD)	297	106
**Blood counts prior to first LP session**		
Mean leukocytes /nl (+/- SD)	27	20
Mean PB CD34^+^ cells at first LP/µl (+/- SD)	85	89
**First LP session**		
Mean collected CD34^+^cells x10^6^/kg (+/- SD)	7,4	5
**Overall PBSC collection result**		
Mean CD34^+^cells x10^6^/kg (+/- SD)	7,9	4,6
**LP sessions**		
Median, n (range)	1	(0–3)
Patients with distinct number of LP sessions		
1	153	86
2	24	13
3	1	1
Terminated prior to LP	2	1
**Median time from mobilization to ASCT (months**,** range)**	1	(1–26)
**HIGH DOSE CHEMOTHERAPY**		
Melphalan dose		
200mg/m^2^	150	83
170mg/m^2^	7	4
150mg/m^2^	4	2
140mg/m^2^	7	4
100mg/m^2^	6	3
Not applied	6	3
**Autologous stem cell graft**		
Mean CD34^+^cells x10^6^/kg (+/- SD)	5,3	3,4

### PBSC mobilization and collection

PBSC mobilization with chemotherapy and collection were conducted on an inpatient basis. Patients were mobilized using the CAD regimen (cyclophosphamide 1000 mg/m²/day on day 1, doxorubicin 15 mg/m²/day on days 1 to 4, and dexamethasone 40 mg/day orally on days 1 to 4) or the CYCLO regimen (cyclophosphamide 1000 mg/m²/day on day 1 and 2, and dexamethasone 20 mg/day orally on days 1 to 4). G-CSF was applied at 10 µg/kg for at least 4 consecutive days. In patients undergoing chemo-free mobilization after 2023, an outpatient mobilization approach with on-demand plerixafor was performed: Patients applied G-CSF at 10 µg/kg for three days. On day four, CD34 count in the PB was measured. In poor mobilizers, plerixafor was applied at 0.24 mg/kg bw on day four and LP was initiated on day five.

A peripheral or central venous catheter was used for stem cell collection. The maximum collection time per session was limited to five hours. A maximum of four times the patient’s total blood volume could be processed. Consecutive daily LP sessions continued until the collection target was met or additional sessions were deemed unlikely to yield sufficient cells due to low PB CD34^+^ counts or inadequate CD34^+^ cell yields. The number of CD34^+^ cells collected was determined by flow cytometry. The collected PBSCs were cryopreserved with dimethylsulfoxide and stored below − 130 °C.

### High-dose chemotherapy and ASCT

Patients underwent ASCT after a median time of one month after LP (range 1–26 months). Over 90% of patients underwent ASCT as part of first-line therapy. All but one patient received high dose melphalan (HDM) as conditioning regimen, with a total dose of 200 mg/m² applied on days − 3 and − 2. The melphalan dose was reduced to 85%, 75%, 70%, and 50% for patients with a creatinine clearance of ≤ 60 mL/min, ≤ 45 mL/min, ≤ 30 mL/min, and ≤ 10 mL/min, respectively. A minimum of 2 × 10⁶ CD34^+^ cells/kg was reinfused on day 0, accompanied by standard supportive therapy (500 mg acetaminophen orally, 2 mg clemastine intravenously, and 10 mg dihydrocodeine orally), within 10 min of thawing PBSC at 37 °C. For antibiotic and antiviral prophylaxis, patients received trimethoprim-sulfamethoxazole 960 mg orally thrice per week for 3 months, and acyclovir 400 mg orally twice daily for 6 months following ASCT. To facilitate leukocyte reconstitution, G-CSF was applied at 5 µg/kg from day + 1 until leukocyte reconstitution in patients transplanted after 2016.

After ASCT, daily blood counts were performed until leukocyte and platelet engraftment occurred. Leukocyte engraftment was defined as a leukocyte count of ≥ 1 × 10⁹/L. Platelet engraftment was defined as the first day platelets reached 20 × 10⁹/L or 50 × 10⁹/L without the need for platelet transfusion. To assess long-term stem cell function, platelet counts were also evaluated one year after ASCT.

### Statistical analysis

Descriptive statistics were conducted using SPSS (v. 29). The data are presented as absolute values and percentages, medians with ranges, or means with standard deviations (SDs). Categorical variables were compared using the chi-square test. Group means for continuous variables were compared using analysis of variance (ANOVA) with Bonferroni correction as needed. The Kruskal–Wallis test was used to compare the median values of non-normally distributed variables. Normality was assessed using the Shapiro-Wilk test. Analyses were performed on the whole cohort unless stated otherwise. Logistic regression analysis was conducted using SPSS. A p-value of < 0.05 was considered statistically significant.

## Results

### Patient characteristics

In total, 180 consecutive patients with newly diagnosed AL amyloidosis which underwent autologous stem cell collection between 2008 and 2024 at our center were included in this analysis. Detailed patient characteristics are listed in Table 1. The median age at diagnosis was 55 years (range 36–69), with 62% of patients being male. 35% had as underlying disease a smoldering myeloma, and 19% multiple myeloma, and 10% had clinically symptomatic disease with bone lesions or anemia. Lambda was the involved light chain in 73% of patients. Organ involvement was noted in 48% of patients for the heart and 60% for the kidney. 16% of patients exhibited liver involvement, and 20% involvement of the gastrointestinal tract. For induction, 51% received a bortezomib-based regimen (e.g., CyBorD, BorD), 15% were treated with a daratumumab-containing regimen, and 27% underwent chemotherapy-free induction with glucocorticoids only. 7% were treated with other, mostly lenalidomide- or melphalan-based, regimens. The median number of induction cycles was 3 (range 0–15). Chemo-free mobilization with G-CSF was performed in 23% of patients, while 39% received cyclophosphamide and dexamethasone (CYCLO), and 33% received cyclophosphamide, adriamycin, and dexamethasone (CAD). Patients underwent a median of 1 LP session (range 1–3). Overall, 178 out of 180 patients that were subjected to (chemo-)mobilization underwent stem cell collection and collected at least 2 × 10^6^/kg. One patient did not finalize stem cell collection due to kidney failure in 2009, which was attempted with CYCLO after 4 cycles of induction with thalidomide, cyclophosphamide, and dexamethasone. Another patient developed sepsis after mobilization chemotherapy with CAD in 2008, which was conducted after repeated dexamethasone pulse therapy. Both patients recovered from the adverse events and were subjected to alternative therapies thereafter. Mean overall collection result was 7.9 (+/- 4.6) x10^6^/kg in the whole cohort. Eventually, 175 out of 178 patients proceeded to HDM/ASCT.

### Induction therapies affect stem cell yield in AL amyloidosis

**Fig. 1:**
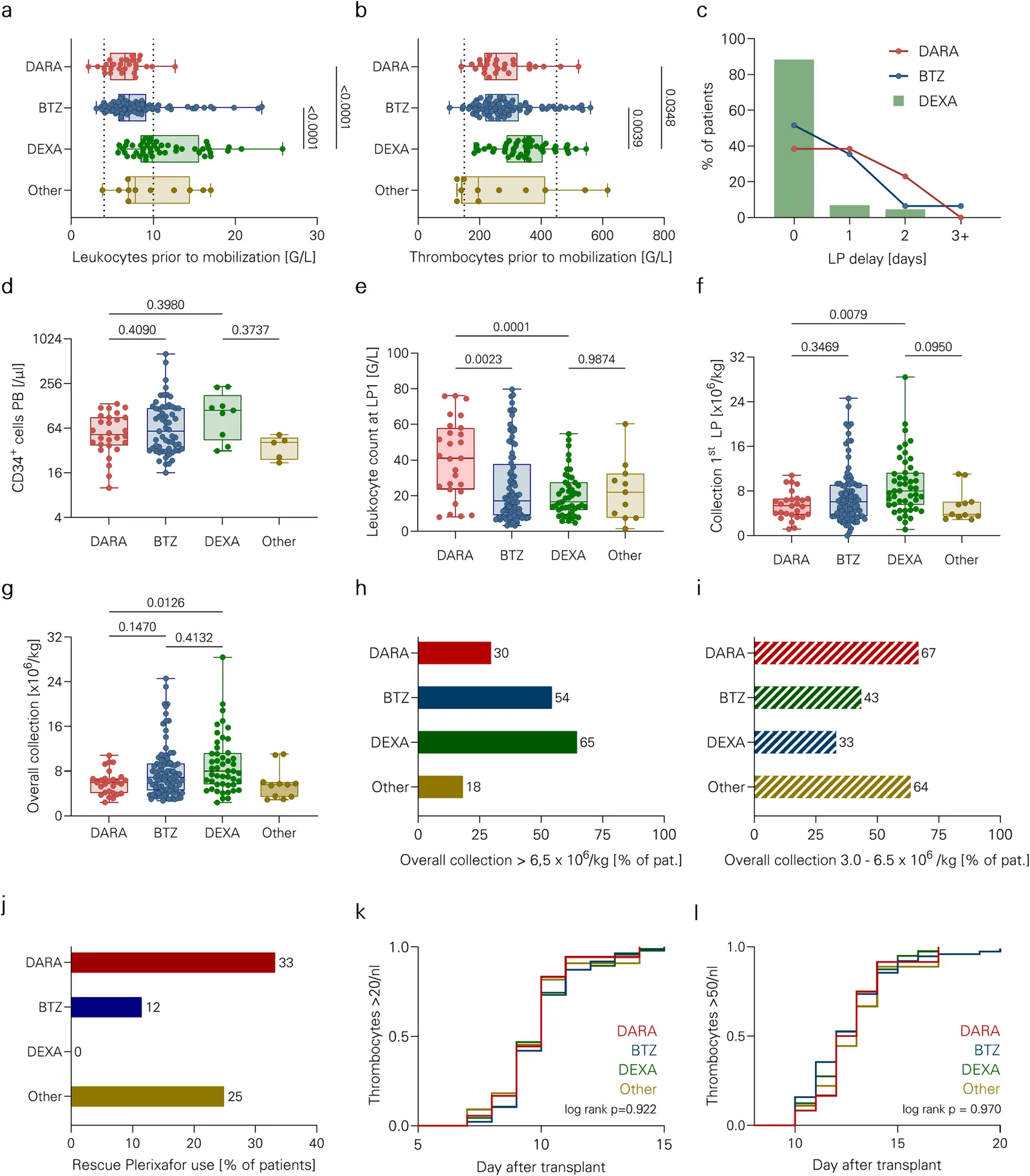
Stem cell mobilization and collection metrics after different induction therapies. **a** Leukocyte counts prior to mobilization therapy are depicted (Dot plots and Boxplots with min/max whiskers, ANOVA. DARA, daratumumab-containing; BTZ, bortezomib containing, DEXA, dexamethasone containing). **b** Thrombocyte counts after different induction therapies are depicted (ANOVA.) **c** Leukapheresis (LP) delays surpassing the scheduled first collection day are shown for the respective induction groups as percentage of each group. **d** CD34+ cell counts (/µl) in the peripheral blood on the first scheduled collection day are shown (n = 101, ANOVA). **e** Leukocyte counts prior to the first LP session are depicted (ANOVA). **f** Collected CD34+ cells x 106 per kilogram body weight (thereafter: x106/kg) at the first LP session (LP1) are depicted (ANOVA). **g** Overall CD34+ cell collection results are depicted (ANOVA). **h** The fraction of patients that collected > 6.5 × 10⁶/kg is shown. **i** The fraction of patients that collected > 3 - <6.5 × 10⁶/kg is shown. **j** The share of eligible patients that underwent rescue mobilization with plerixafor is shown. **k** Thrombocyte reconstitution (> 20/nl) after different induction therapies is depicted (n = 169). **l** Thrombocyte reconstitution (> 50/nl) after different induction therapies is shown (n = 148)

Patients underwent different induction treatments prior to stem cell collection. Daratumumab-based regimen (DARA, e.g., DaraCyBorD), bortezomib-based regimen (BTZ, e.g., CyBorD), dexamethasone only (DEXA) and others (e.g., Melphalan-Dex, Thalidomide-Dex, Lenalidomide-Dex) were applied. Pretreatment affected pre-mobilization leukocyte and thrombocyte counts. DARA- and BTZ-treated patients exhibited lower leukocyte counts versus DEXA-treated patients (6.5 versus 11.5/nl, *p* < 0.001, Fig. 1a, and 8.2 versus 11.5/nl, *p* < 0.0001). Likewise, BTZ-treated and DARA-treated patients exhibited lower thrombocyte levels prior to mobilization when compared to DEXA-treated patients (283 and 278 versus 345/nl, *p* = 0.0042, Fig. 1b). The introduction of BTZ and DARA during induction led to delayed mobilization (LP delay) in 48% and 62% of patients, respectively. After DARA, 23% of patients had an LP delay of > = 2 days (Fig. 1c). Since 2014, flow cytometric analyses of CD34 counts in the peripheral blood (PB) were recorded. No differences in CD34^+^ levels at the first leukapheresis (LP1) were observed between groups (*p* = 0.175, Fig. 1d). Leukocyte counts at LP1 were significantly higher in DARA-treated patients (*p* = 0.002, Fig. 1e). In contrast, CD34 collection at LP1 was significantly lower in DARA-treated patients when compared to the DEXA group (5.44 versus 9.13, *p* = 0.008, Fig. 1f). No such difference was observed between the other groups. In 2009, rescue mobilization with plerixafor was established at our institution. Despite plerixafor being available, overall CD34 cell collection was lower in the DARA group compared to DEXA-treated patients (5.93 versus 9.24, *p* = 0.013). No significant difference was observed between the BTZ and DEXA groups (8.01 versus 9.24, *p* = 0.413, Fig. 1g). According to our analysis of patients that underwent stem cell mobilization between 1998 and 2007, good mobilizers were defined as patients that collected 6,5 × 10^6^/kg or more stem cells [23]. In this previous analysis, high stem cell doses were associated with faster leukocyte reconstitution and higher thrombocyte counts one year after HDM/ASCT. The percentage of patients collecting at least 6,5 × 10⁶/kg were reduced after BTZ versus DEXA (54% versus 65%). DARA exposure further reduced the stem cell yield such that only 30% of patients were good mobilizers (Fig. 1h). Consequently, 67% of patients with DARA only collected intermediate dose stem cell products (> 3.0 but < 6.5 × 10^6^/kg, Fig. 1i). Rescue mobilization was available for patients treated with BTZ or DARA. While plerixafor was applied to only 12% of eligible patients in the BTZ group, 33% of DARA-treated patients had to be subjected to rescue mobilization (Fig. 1j). Thrombocyte reconstitution to 20/nl and 50/nl after ASCT was not affected by the use of DARA. More than 50% of patients exhibited thrombocytes of 20/nl and 50/nl on day 10 and 12 after autologous transplantation, respectively (Fig. 1k and l). Leukocyte reconstitution to 0.5 and 1.0 G/L in patients receiving post-transplant G-CSF was not affected by induction therapy (9 days and 10 days, *p* = 0.930 and 0.879, respectively, Supplementary Figure S1A-B). Thrombocyte counts one year after ASCT, however, were significantly higher in DEXA-treated patients (median 266/nl) compared to BTZ- and DARA-treated patients (216/nl, *p* = 0.008 and 210/nl, *p* = 0.044, respectively) as shown in Supplementary Figure S1C.

### Mobilization chemotherapy incorporating an anthracycline improves stem cell collection

**Fig. 2:**
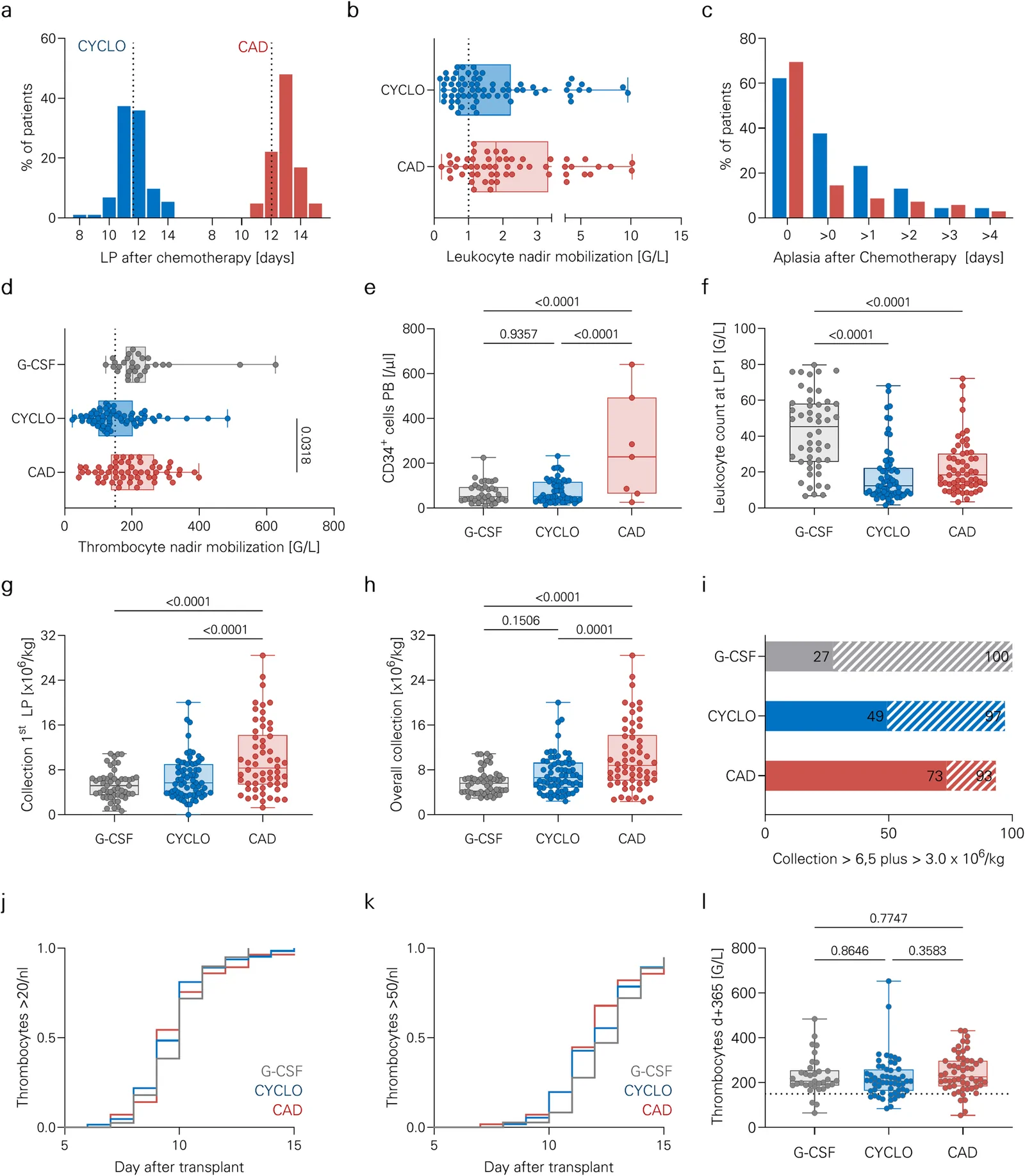
Different mobilization strategies impact stem cell collection outcomes. **a** Distinct duration of mobilization (from beginning of mobilization chemotherapy to LP1) is depicted. Patients were grouped according to mobilization chemotherapy received: cyclophosphamide (CYCLO, n = 70) or cyclophosphamide, adriamycin, dexamethasone (CAD, n = 59). Dotted lines indicate the planned LP date according to SOPs. **b** The lowest leukocyte count (nadir) during different mobilization chemotherapies is shown (n = 123). The dotted line indicates a leukocyte count of 1/nl (Student’s t test). **c** Aplasia duration (leukocytes < 0.5/nl) after mobilization chemotherapy is shown. **d** The lowest thrombocyte count during mobilization is depicted (n = 125). The dotted line indicates a value of 150/nl. **e** CD34+ cell counts in the peripheral blood (PB) at LP1 are depicted (n = 101, ANOVA). **f** Leukocyte counts in PB at LP1 after different mobilization strategies are shown (ANOVA). **g** Collected CD34+ (x106/kg) at the first LP session (LP1) are depicted (ANOVA). **h** Overall CD34+ cell collection results are depicted (ANOVA). **i** The fraction of patients that collected > 6.5 × 10⁶/kg (solid bar) or 3.0-6.49 × 10⁶/kg (hatched bar) are shown. **j** Thrombocyte reconstitution (> 20/nl) according to days after transplant is depicted (n = 169). **k** Thrombocyte reconstitution (> 50/nl) according to days after transplant is depicted (n = 148). **l** Thrombocyte counts approximately 365 days after transplant are shown (n = 145 ,ANOVA)

Most patients were subjected to mobilization chemotherapy with either cyclophosphamide (CYCLO) or cyclophosphamide, Adriamycin, and dexamethasone (CAD). A small portion of patients underwent chemo-free mobilization with G-CSF without plerixafor. Planned LP1 dates for CYCLO and CAD were 12 and 13 days after the start of chemotherapy, respectively. In the CYCLO group, 16% of patients showed an LP delay versus 22% in the CAD group (Fig. 2a). Likewise, 48% of patients in the CYCLO group were subjected to LP prior to the planned LP date. Patients in the CYCLO group showed higher rates of leukopenia < 1 /nl (39% versus 18%, Fig. 2b). Aplasia as defined by leukocyte count < 1/nl longer than 24 h was seen in 38% (CYCLO) versus 30% of patients, respectively (Fig. 2c). CYCLO led to a significantly lower thrombocyte nadir (median 166/nl versus 197/nl, *p* = 0.0318) compared to CAD (Fig. 2d). In contrast, CD34^+^ counts in the PB were significantly higher in evaluable CAD patients (260 versus 74, *p* < 0.0001, Fig. 2e). While G-CSF led to higher total leukocyte counts at LP1 than CAD (40 versus 23/nl, *p* < 0.0001, Fig. 2f), this did not reflect higher numbers of CD34 cells in the PB (61/nl versus 74/nl, *p* = 0.7849). More potent CD34 cell mobilization with CAD translated in an increased CD34 cell yield at LP 1 compared to CYCLO (10.02 versus 6.54, *p* < 0.0001) and G-CSF (5.08, *p* < 0.0001, Fig. 2g). Similarly, CAD resulted in higher overall CD34 cell collection after all LP sessions compared to G-CSF (10.41 versus 5.54, *p* < 0.0001). No significant difference was seen between CYCLO and G-CSF groups (7.29 versus 5.54, *p* = 0.151, Fig. 2h). Eventually, CAD mobilization led to collection of > 6.5 × 10^6^/kg in 73% of patients. Successful collection of > 6.5 × 10^6^/kg was feasible in 49% of patients that received CYCLO and in 27% after G-CSF without plerixafor. Thrombocyte reconstitution after autologous transplantation did not significantly differ between groups (median 9–10 days for 20/nl and 12–13 days for 50/nl, Fig. 2j-k). Similarly, thrombocyte counts one year after ASCT did not differ between groups (*p* = 0.707, Fig. 2l).

### Univariate and multivariate analyses of factors influencing collection outcomes

To compare patient characteristics between poor to intermediate mobilizers with good mobilizers, patients were grouped by the cumulative number of CD34^+^ cells collected in all LP sessions. In our previous cohort including patients from 1998 to 2007, stem cell doses > 6.5 × 10⁶/kg were associated with favorable outcomes such as shorter time to reconstitution and lower rates of long-term thrombocytopenia. The first group was comprised of good mobilizers (*n* = 91, > 6.5 × 10⁶/kg), the second group of poor to intermediate mobilizers (*n* = 88, 2-6.49 × 10⁶/kg). Univariate analyses were performed (Table 2). Baseline characteristics such as gender, age and underlying diagnosis were not significantly different between groups (Table 3). There was a trend to a higher percentage of kappa as the amyloidogenic light chain in intermediate and poor mobilizers (33% versus 21%, *p* = 0.091). Interestingly, patients with heart involvement were more likely to show intermediate or poor mobilization (59% versus 37%, *p* = 0.006). On the other hand, kidney involvement correlated with good mobilization outcomes (68% versus 51%, *p* = 0.021). In accordance with the results above, induction groups were significantly associated with different collection outcomes. In particular, DARA-exposed patients comprised 22% of intermediate and poor mobilizers, but only 9% of good mobilizers (*p* = 0.021). Mobilization regimens were significantly associated with collection outcome, with CAD inducing good mobilization (46% versus 18%) and G-CSF without plerixafor indicating poor or intermediate collection outcomes (35% versus 11%). Interestingly, pre-mobilization leukocyte counts, but not thrombocyte counts were associated with good mobilization (10/nl versus 8/nl). In poor mobilizers, rescue plerixafor was applied in 17% of patients compared to 5% in good mobilizers (*p* = 0.017). Other relevant collection outcomes such as collected CD34^+^ cells during LP1 and CD34 counts in peripheral blood at LP1 were significantly different between groups.

**Table 2:** Patient characteristics stratified by mobilization outcomes. ^a^ Data available in *n* = 100 patients G-CSF, granulocyte colony-stimulating factor; GI, gastrointestinal; IMiD, immunomodulatory drugs; LP, leukapheresis; MGUS, monoclonal gammopathy of undetermined significance; MM, multiple myeloma; SD, standard deviation;

	Good mobilizer		Intermediate and poor mobilizer		
	*n*	%	*n*	%	*p* (univariate)
**Patient number**,** n**	91	100	88	100	
**Gender**,** n (%)**					
Male	54	59	58	66	0.440
Female	37	41	30	34	
**FIRST DIAGNOSIS**					0.878
MM	19	21	16	18	
MGUS	40	44	41	47	
Smouldering	32	35	30	34	
other	0	0	1	1	
**Median age at diagnosis**,** years (range)**	54	(36–69)	55	(41–68)	0.759
**Mean age at diagnosis**,** years (SD)**	54	7	55	7	0.487
**Light chain type**,** n (%)**					
Lambda	72	79	59	67	0.091
Kappa	19	21	29	33	
**Organ involvement**					
Heart	34	37	52	59	**0.006**
Kidney	62	68	45	51	**0.021**
Liver	16	18	11	13	0.406
Peripheral nervous system	11	12	10	11	1.000
Autonomous nervous system	8	9	7	8	1.000
GI tract	17	19	19	22	0.852
soft tissue	42	46	33	38	0.228
**INDUCTION TREATMENT**					
**First line treatment - exposure to agents**					
Daratumumab-exposed	8	9	19	22	**0.021**
Bortezomib-exposed	56	62	62	70	0.27
Melphalan-exposed	1	1	3	3	0.362
**Induction group**					**0.002**
Daratumumab-based (e.g., DaraCyBorD, DaraBorD)	8	9	19	22	
Bortezomib-based (CyBorD, BorD)	50	55	42	48	
Chemotherapy-free (No treatment, glucocorticoids)	31	34	17	19	
Other (e.g., IMiD, melphalan)	2	2	10	11	
**Median number of cycles of induction and maintenance (range)**	3	(0–12)	3	(0–15)	0.064
**PBSC MOBILIZATION/COLLECTION**					
**Mobilisation regimen**					**< 0.001**
Cyclophosphamide, adriamycin, dexamethasone (CAD)	42	46	16	18	
Cyclophosphamide, dexamethasone	35	38	35	40	
G-CSF only	10	11	31	35	
G-CSF + pre-emptive plerixafor	4	4	6	7	
**Rescue Plerixafor**					**0.017**
Yes	5	5	15	17	
No	86	95	73	83	
**Blood counts prior to mobilization**					
Mean leukocytes /nl (+/- SD)	10	5	8	3	**0.013**
Mean Thrombocytes /nl (+/- SD)	312	105	281	105	0.655
**Blood counts pre first LP session**					
Mean leukocytes /nl (+/- SD)	25	19	28	20	0.554
Mean PB CD34^+^ cells at first LP/µl (+/- SD)^a^	126	113	51	38	**0.002**
**First LP session**					
Mean collected CD34^+^cells x10^6^/kg	10,4	5	4,3	1	**< 0.001**
**Overall PBSC collection result**					
Mean CD34^+^cells x10^6^/kg (+/- SD)	11,0	4,5	4,7	1,2	**< 0.001**
**LP sessions**					
Median, n (range)	1	(1–2)	1	(1–3)	0.602
Patients with distinct number of LP sessions					0.586
1	80	88	74	84	
2	11	12	13	15	
3	0	0	1	1	

**Table 3:** Multivariable logistic regression analysis. CAD, Cyclophosphamide, adriamycin, dexamethasone; CI, confidence interval; G-CSF, granulocyte colony-stimulating factor;

**Variable**	**Overall CD34**^+^ **collection results**		**Plerixafor use**	
	**(≥ 6.5 × 10** ^6^ **/kg bw versus < 6.5)** ^a^		**(yes versus no)** ^b^	
	Odds ratio	p value	Odds ratio	p value
	[95% CI]		[95% CI]	
Light chain (lambda versus kappa)	0.62	0.206	1.11	0.872
	[0.29–1.31]		[0.39–7.18]	
Daratumumab-based induction (yes versus no)	0.49	0.157	22.5	**< 0.001**
	[0.18–1.32]		[7.0-72.6]	
Mobilization type (G-CSF only versus other)	0.31	**0.009**		/
	[0.13–0.75]			
Mobilization type (CAD versus other)	2.27	**0.044**		/
	[1.02–5.01]			
Number of involved organs (≥ 3 versus < 3)	1.18	0.645	1.97	0.262
	[0.59–2.37]		[0.60–6.40]	
Number of prior treatment cycles (≥ 3 versus < 3)	0.87	0.703	1.13	0.857
	[0.42–1.80]		[0.31–4.09]	

^a^ number of patients with complete data set, *n* = 178 ^b^ number of eligible patients, *n* = 156

In a second step, multivariable logistic regression analyses were performed. To this end, characteristics that correlated with collection outcomes in univariate analysis were used as dependent variables. These include light chain type (lambda versus kappa), daratumumab induction (yes versus no), mobilization type (G-CSF versus other), mobilization type (CAD versus other), number of involved organs (≥ 3 versus < 3), and number of prior treatment cycles (≥ 3 versus < 3). Endpoints were overall collection results (≥ 6.5 versus < 6.5 × 10⁶/kg) and plerixafor use (yes versus no) in *n* = 156 eligible patients. Lambda light chain (Odds ratio 0.62, 95% Confidence interval (CI) 0.29–1.31, *p* = 0.206) and daratumumab induction (0.49, 0.18–1.32, *p* = 0.157) showed a trend towards poor and intermediate mobilization. Mobilization with G-CSF without plerixafor significantly correlated with inferior collection outcomes (0.31, 0.13–0.75, *p* = 0.009). In contrast, CAD mobilization was superior to all other regimen (2.27, 1.02–5.01, *p* = 0.044). The other tested variables did not correlate with overall CD34^+^ cell collection. Likewise, only daratumumab induction was significantly associated with the need for rescue plerixafor (22.5, 7.0-72.6, *p* < 0.001), while light chain type, number of involved organs and number of treatment cycles prior to mobilization were not.

### Higher stem cell doses at autologous transplantation reduce short-term and long-term thrombocytopenia

**Fig. 3:**
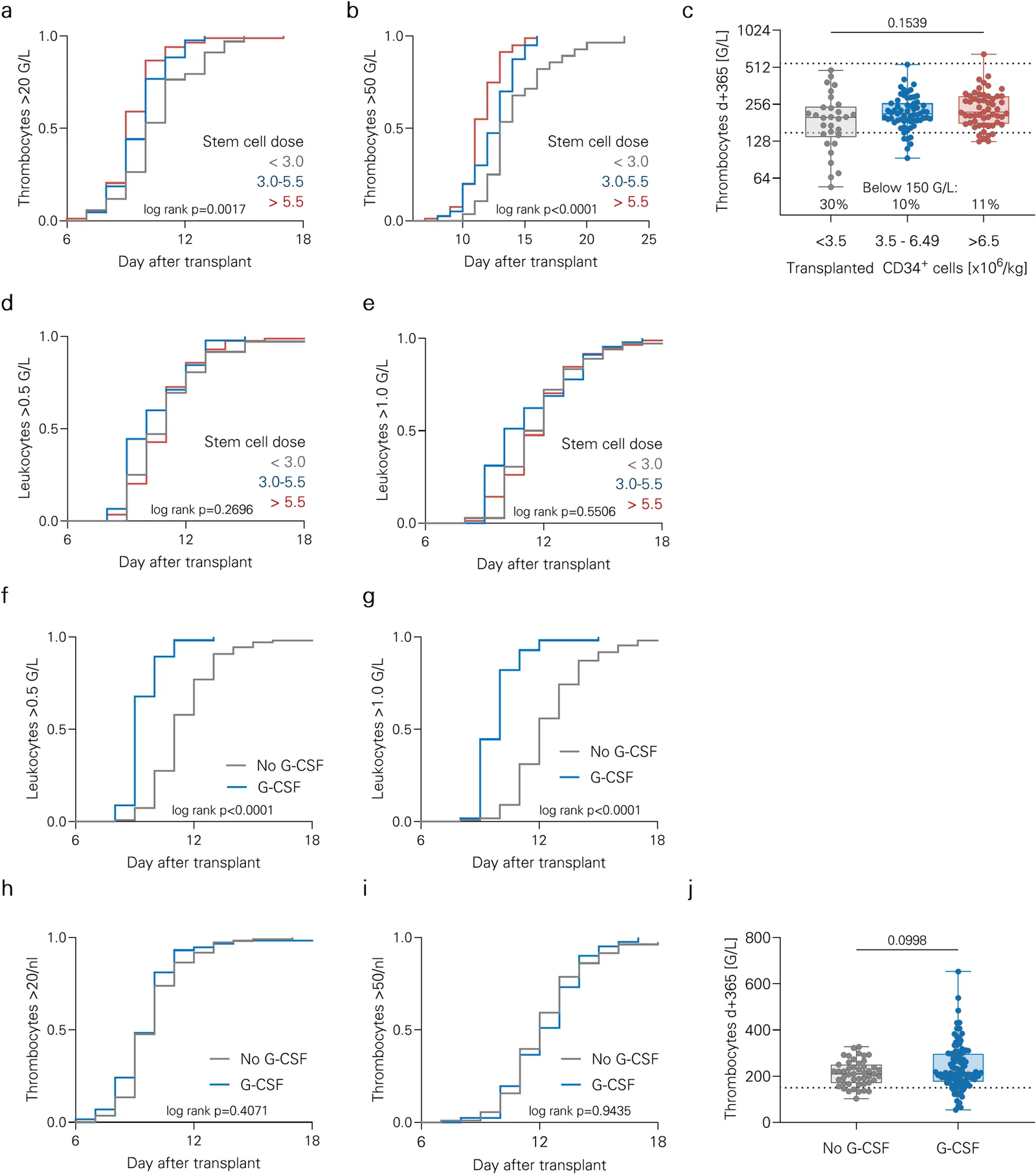
Stem cell doses and G-CSF application affect short-term and long-term hematopoietic reconstitution. **a**-**b** Thrombocyte reconstitution (a) > 20/nl, n = 169 b) > 50/nl, n = 148) after transplant in patients grouped by stem cell dose is depicted. **c** Thrombocyte counts one year after transplantation in patients grouped by stem cell dose is shown (n = 145, ANOVA). **d**-**e** Leukocyte reconstitution ((d) > 0.5/nl, n = 168, e) > 1.0/nl, n = 165) after transplant in patients grouped by stem cell dose is depicted. **f**-**g** Leukocyte reconstitution ((f) > 0.5/nl, n = 168, g) > 1.0/nl, n = 165) after ASCT with or without G-CSF is depicted. **h**-**i** Thrombocyte reconstitution (h) > 20/nl, n = 169, i) > 50/nl, n = 148) after transplant with or without G-CSF is depicted. **j** Thrombocyte counts one year after transplantation with or without post-transplant G-CSF are shown (n = 145)

In our previous analysis of patients transplanted between 1998 and 2007, stem cell doses > 6.5 × 10⁶/kg were associated with faster reconstitution and higher thrombocyte counts at one year after HDM/ASCT. However, since the introduction of quadruplet induction, stem cell doses over 6.5 are rarely reached. Accordingly, patients were grouped by applied stem cell doses: ≤ 3.5 (low *n* = 41), 3.5–6.49 (intermediate, *n* = 79), or > 6.5 × 10^6^/kg (high, *n* = 60). Patients that received high stem cell numbers showed a shorter time to thrombocyte reconstitution > 20/nl (median time 9 days versus 10 days, *p* = 0.002, Fig. 3a). Furthermore, patients with low or intermediate stem cell doses exhibited significantly prolonged thrombocyte reconstitution > 50/nl (day + 13 versus day + 11, *p* < 0.001, Fig. 3b). To evaluate the effects of stem cell doses on long-term bone marrow function, thrombocyte counts at 12 months post transplantation were compared between groups. Patients that underwent any antineoplastic treatment within the first year after ASCT were excluded. Patients that received high stem cell doses showed numerically, but not significantly higher thrombocyte counts 12 months post-transplantation compared to the low stem cell dose group (245/nl versus 207/nl, *p* = 0.128, Fig. 3c). Furthermore, low stem cell doses were associated with a higher rate of thrombocytopenia up to 30% compared to 10–11% after intermediate and high stem cell doses. No significant differences were observed between groups regarding leukocyte reconstitution (day 10–11 for 0.5G/L, day 10–12 for 1.0G/L, Fig. 3d-e). In 2017, post-ASCT G-CSF application became institutional standards for all patients. With G-CSF, leukocyte reconstitution was significantly shortened: Median reconstitution to > 0.5 G/l was reached on day 9 versus 11 (*p* < 0.001, Fig. 3f) and reconstitution to 1.0 G/l on day 10 versus 12 (*p* < 0.001, Fig. 3g). Thrombocyte reconstitution was not affected by G-CSF application with reconstitution to 20/nl at day ten and to 50/nl at day twelve for both groups (Fig. 3h-i). G-CSF did not lead to a significantly different thrombocyte count at 1 year (239 versus 213, *p* = 0.100, Fig. 3j).

### Mobilization with rescue and preemptive plerixafor

**Fig. 4:**
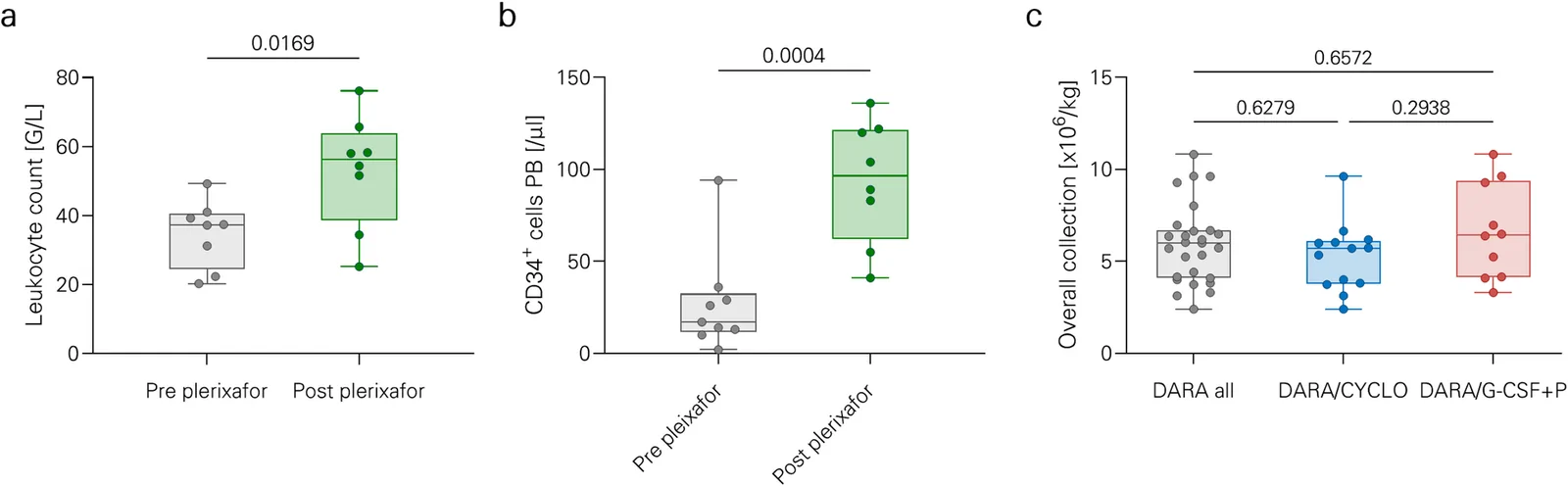
Successful stem cell mobilization with G-CSF and plerixafor. **a** Leukocyte counts prior to and after plerixafor application in patients treated with DARA-based induction therapy and mobilized with G-CSF (n = 27). **b** CD34+ counts in the PB of patients before and after treatment with plerixafor after G-CSF mobilization (n = 8). **c** Overall CD34+ collection outcomes of patients treated with DARA (n = 27) and either chemomobilization or G-CSF with plerixafor (ANOVA)

In 2009, rescue mobilization with plerixafor was established at our institution. Since DARA-treated patients exhibited lower CD34 cell counts in the peripheral blood at the intended first collection day, these patients underwent rescue mobilization with plerixafor more often (33% versus 12% after BTZ, Fig. 1j). As shown earlier, CAD mobilization was effective, thus no patients treated with CAD underwent plerixafor treatment. Leukocyte counts were significantly increased upon plerixafor application (34.8/nl versus 53.0/nl, *p* = 0.017, Fig. 4a). Plerixafor application significantly augmented CD34 mobilization from 26.8/µl before plerixafor to 93.8/µl after application (*p* = 0.0004, Fig. 4b). This resulted in improved CD34 collection without collection failures or low stem cell doses occurring after plerixafor application. Plerixafor was well-tolerated and did not lead to (prolonged) hospitalization. In 2024, outpatient mobilization with G-CSF and plerixafor was introduced. All nine patients underwent induction therapy with DARA and were subjected to G-CSF from day + 1 to day + 4. Only at CD34^+^ counts of > 50 in the PB, LP was initiated on day + 4. Only one out of nine patients reached the cut-off with a CD34 count of 94/µl. For all other patients, application of plerixafor at 0.24 mg / kg bw was performed on day + 4, followed by LP on day five. Overall collection after G-CSF was comparable to patients that underwent DARA induction followed by inpatient chemomobilization with CYCLO and G-CSF (6.64 versus 5.26/nl, *p* = 0.294, Fig. 4c).

## Discussion

Autologous stem cell collection in AL amyloidosis is a prerequisite for high-dose chemotherapy and autologous stem cell transplantation. Anti-CD38 antibodies significantly improved response rates and survival in AL amyloidosis. However, their impact on stem cell collection outcomes have been unclear. We here report on stem cell collection metrics in AL amyloidosis patients including those treated with up-to-date triplet and quadruplet regimen.

While HDM/ASCT has been the leading therapeutic modality for younger patients, its role is declining. The addition of daratumumab to CyBorD significantly increased hematological complete remission rates in AL amyloidosis from 19.2% to 59.5% [3]. In patients achieving CR, most centers omit consolidation with HDM/ABCT [8]. However, for low-risk patients with unsatisfactory response (e.g., not achieving hematological VGPR with DaraCyBorD) HDM/ASCT is a preferred treatment option [4, 8, 11, 32]. Patients harboring gain 1q21 show adverse outcomes when treated with DaraCyBorD [33–35], while the prognostic effect of t(11;14) for bortezomib treated patients seems to be neutralized with up-to-date induction treatment [36]. In carefully selected patients, outcomes after heart transplantation and subsequent HDM/ASCT are also promising [15]. In this cohort, only in two out of 178 patients, mobilization therapy was abrupted due to adverse events. Likewise, only three out of 175 patients did not undergo HDM/ASCT despite successful stem cell collection. With careful patient selection, unsuccessful attempts for stem cell collection and transplantation are rare in our center. Of note, patient selection based on HDM/ASCT eligibility explains the relatively low proportion of patients with heart involvement in our cohort compared to the whole population of patients with newly diagnosed AL amyloidosis.

Quadruplet therapy including anti-CD38 antibodies is the standard-of-care in transplant-eligible multiple myeloma patients [37–39]. Several reports show that anti-CD38 have a negative impact on stem cell yield in those patients [29–31]. The application of plerixafor might curb this effect, but is not able to fully compensate it [40]. In contrast to multiple myeloma, tandem transplantation in AL amyloidosis is used rarely, thus, the collection of a single stem cell product might be sufficient. However, daratumumab exposure was associated with a low proportion of patients collecting high dose stem cell products in our cohort. Daratumumab exposure showed a trend to impaired collection in the multivariate analysis without reaching significance. This might be due to the preemptive application of plerixafor that was introduced at our institution in 2024 as well as the option to use plerixafor for rescue mobilization. In contrast, mobilization with G-CSF and CAD were significantly associated with collection outcomes in the multivariable logistic regression analysis. This suggests that the choice of mobilization therapy has a stronger impact than induction therapy. However, the choice of mobilization strategy was not randomized in our cohort, thus introducing bias. Foremost, patients with known cardiac involvement did not undergo CAD chemotherapy, which might explain the lower collection yield in this population. Leukocyte counts and thrombocyte counts after induction therapy were associated with collection outcomes in the univariable analysis. While higher leukocyte counts after DEXA might be due to glucocorticoid activity, thrombocytopenia might be a marker for early bone marrow impairment. Further research should focus on predictive markers for short or long-term bone marrow function.

In contrast to other centers, we relied on mobilization chemotherapy prior to stem cell collection until up to 2023. Mobilization chemotherapy has certain advantages. As described previously as well as in this work, chemotherapy increases stem cell yield [23, 41, 42]. In patients with borderline eligibility, inpatient chemomobilization might be a suitable test run prior to HDM/ASCT. Our detailed analyses support the use of chemomobilization in patients for whom plerixafor is not available.

Chemomobilization also has certain disadvantages. Neutropenic fever can become a life-threatening adverse reaction in patients treated with chemotherapy for mobilization, potentially delaying consolidation with HDM/ASCT. In our cohort, however, no grade IV or V adverse events were seen with chemomobilization. Furthermore, the addition of adriamycin in the CAD regiment significantly improved outcomes compared to CYCLO. However, this regimen should not be applied to patients with suspected or proven heart involvement, thus limiting its use in AL amyloidosis. The overall toxicity profile and patient burden associated with chemomobilization does not justify its use anymore in patients for whom plerixafor is available.

At our institution, the choice of mobilization regimen has changed. Data ranging from 1998 to 2007 showed high collection rates after chemomobilization with ifosfamide/G-CSF (19 × 10⁶/kg per LP) and CAD/G-CSF (8.8 × 10⁶/kg per LP). Both regimen are no longer utilized at our center, mostly due to concerns of hemato-, neuro- and cardiac toxicity [23]. The recently used CYCLO/G-CSF regimen results in lower yet sufficient stem cell collection (7.29 × 10⁶/kg overall). Mobilization with G-CSF only is associated with inferior collection outcomes. Lisenko et al. reported a collection of 4.9 × 10⁶ CD34^+^/kg per LP at our center from 1997 to 2007. Other analyses report higher stem cell yields with CYCLO/G-CSF compared to G-CSF only. Perotti et al. report an overall collection of 8.9 × 10⁶/kg with CYCLO/G-CSF versus 8.2 × 10⁶/kg with G-CSF only [24].

Plerixafor is a highly effective measure to facilitate stem cell mobilization in both multiple myeloma and amyloidosis, however, it might not be available to patients in low or middle-income countries [43–45]. In patients expected to exhibit poor mobilization, for whom plerixafor is not available, chemotherapy might be a suitable alternative at a reasonable cost. If plerixafor is available, mobilization with G-CSF and plerixafor is effective in patients after quadruplet induction therapy with DARA. This approach constitutes the current standard at our institution. Liu et al. reported successful stem cell collection with G-CSF and plerixafor in 28 patients yielding a median of 6.0 × 10⁶/kg, however, patients were subjected mostly to doublet or triplet induction therapy, which is not the preferred option for transplant-eligible patients [46]. Therefore, to the best of our knowledge, this is the first report on collection outcomes after quadruplet induction with daratumumab in AL amyloidosis patients.

The analysis has certain disadvantages. Due to the low incidence of AL amyloidosis, the analysis spans over almost 15 years, which introduced temporal confounding. Secondly, patients where not randomized to either mobilization protocol, which introduces bias to the groups receiving CAD or CYCLO. Since this analysis represents single-center data, not all cut-offs may be generalizable to other centers. This is true in particular for stem cell dosing, since international consensus guidelines indicate durable hematopoietic reconstitution with stem cell doses of 3.0–5.0 × 10⁶/kg [47, 48]. Furthermore, the availability of plerixafor has greatly increased since it is now off patent, thus further decreasing the role of mobilization chemotherapy. The role of ASCT in AL amyloidosis is declining. Therefore, the US intergroup trial S2213 (NCT06022939) is currently recruiting patients in order to evaluate the benefit of ASCT after quadruplet induction therapy.

While HDM/ASCT in multiple myeloma might be feasible with stem cell products comprising 2 × 10⁶/kg, stem cell doses > 6.5 × 10⁶/kg are associated with improved reconstitution and thrombopoiesis as shown here and previously by us [23, 49, 50]. Second line therapy is evolving in AL amyloidosis, with agents such as venetoclax, bispecific antibodies and chimeric antigen receptor T cells showing promising activity [21, 22, 51, 52]. All beforementioned modalities are associated with cytopenia to a certain extent. For instance, immune effector cell-associated hematotoxicity (ICAHT) and subsequent infections account for a substantial proportion of non-relapse mortality in CAR T cell therapy [53]. Improved bone marrow function in patients transplanted with a higher stem cell dose might be beneficial for patients eventually undergoing those treatments. Furthermore, a stem cell backup might be beneficial for long-term ICAHT. In a recent multicenter analysis, Muchtar et al. observed a significant overall survival benefit in patients transplanted with > 4.5 × 10⁶/kg [54]. Further analyses should assess whether stem cell dosing affects bone marrow function beyond the 12-month checkpoint and cytopenias during subsequent therapy lines.

In summary, stem cell mobilization is feasible in patients treated with DARA-containing quadruplet induction therapies. However, quadruplet induction with DARA reduced the rate of good mobilizers to 30% despite plerixafor being available as rescue therapy. Chemomobilization containing cyclophosphamide and an anthracycline was more effective than mobilization with G-CSF or cyclophosphamide alone. However, if plerixafor was available, a chemo-free approach with G-CSF with G-CSF is feasible and effective. Multivariable logistic regression suggests that the different mobilization strategies have a stronger impact on stem cell collection than induction therapy. Nevertheless, higher stem cell doses should be pursued due to the beneficial effects on short- and long-term bone marrow function.

## Supplementary information

Below is the link to the electronic supplementary material.

Supplementary Material 1

## Data Availability

All data generated or analysed during this study are included in this published article.
