# Supplementary material for: Impact of daratumumab on stem cell collection in patients with AL amyloidosis
Source: BMC Cancer. 2026 Nov 3;26:1403. doi: 10.1186/s12885-026-17227-0 (PMC13753244; doi:10.1186/s12885-026-17227-0)
Supplement: Supplementary file 1 [file 12885_2026_17227_MOESM1_ESM.pdf]

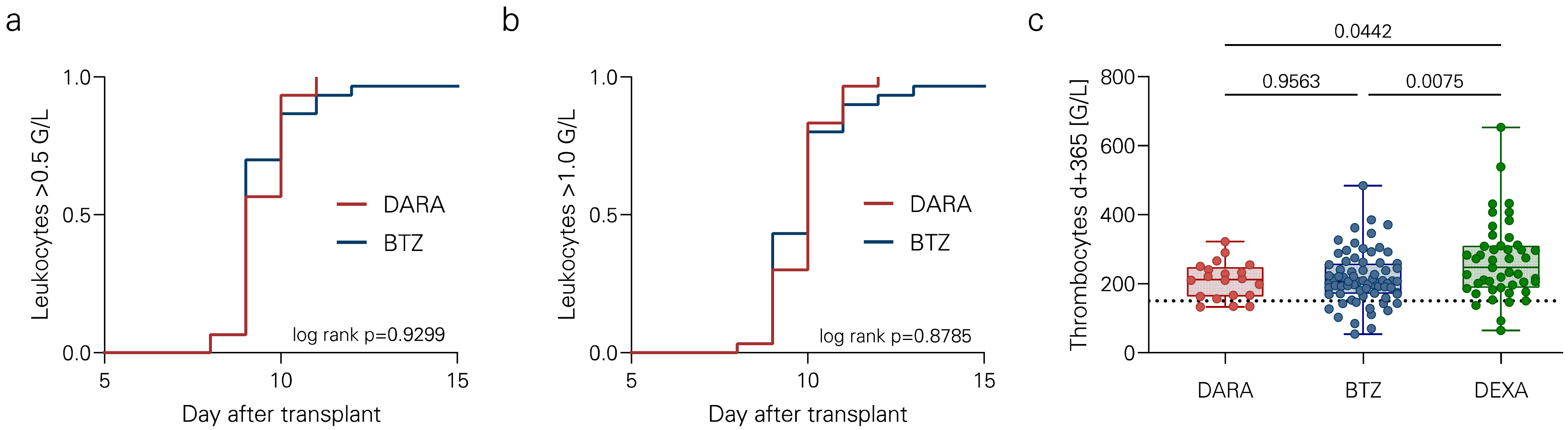

Supplementary Figure S1. Leukocyte reconstitution and thrombocyte counts after different induction therapies. a)-b) Leukocyte reconstitution ((a) >0.5/nl, b) >1.0/nl) after transplant in patients grouped by induction therapy is depicted. n=64 patients who received post-transplant G-CSF were included. c) Thrombocyte counts one year after transplantation after different induction therapies are shown. ANOVA.
